# Neurogenic timing of the inferior olive subdivisions is related to the olivocerebellar projection topography

**DOI:** 10.1038/s41598-023-33497-1

**Published:** 2023-05-02

**Authors:** Yuanjun Luo, Yuhan Chao, Richard Nana Abankwah Owusu-Mensah, Jingyun Zhang, Tatsumi Hirata, Izumi Sugihara

**Affiliations:** 1 Department of Systems Neurophysiology, Graduate School of Medical and Dental Sciences, Tokyo Medical and Dental University, 1-5-45 Yushima, Bunkyo-ku, Tokyo, 113-8519 Japan; 2 Brain Function Lab, National Institute of Genetics, 1111 Yata, Mishima-shi, Shizuoka-ken 411-8540 Japan; 3 Center for Brain Integration Research, Tokyo Medical and Dental University, 1-5-45 Yushima, Bunkyo-ku, Tokyo, 113-8519 Japan

**Keywords:** Development of the nervous system, Neurogenesis, Neuroscience, Anatomy, Nervous system

## Abstract

The olivocerebellar projection is organized into an intricate topographical connection from the inferior olive (IO) subdivisions to the longitudinally-striped compartments of cerebellar Purkinje Cells (PCs), to play an essential role in cerebellar coordination and learning. However, the central mechanisms for forming topography need to be clarified. IO neurons and PCs are generated during overlapping periods of a few days in embryonic development. Therefore, we examined whether their neurogenic timing is specifically involved in the olivocerebellar topographic projection relationship. First, we mapped neurogenic timing in the entire IO by using the neurogenic-tagging system of *neurog2*-CreER (G2A) mice and specific labeling of IO neurons with FoxP2. IO subdivisions were classified into three groups depending on their neurogenic timing range. Then, we examined the relationships in the neurogenic-timing gradient between IO neurons and PCs by labeling topographic olivocerebellar projection patterns and PC neurogenic timing. Early, intermediate, and late groups of IO subdivisions projected to late, intermediate, and early groups of the cortical compartments, respectively, except for a few particular areas. The results indicated that the olivocerebellar topographic relationship is essentially arranged according to the reverse neurogenic-timing gradients of the origin and target.

## Introduction

The inferior olive (IO) located in the ventrocaudal medulla sends climbing fibers to cerebellar Purkinje cells (PCs) to make an essential contribution to cerebellar coordination and learning by controlling their excitability and synaptic responsibility^1,2^. The olivocerebellar projection is characterized by the segregated parallel topography; distinct subdivisions of the IO project to distinct sets of longitudinally-striped compartments (also designated as modules^3^) in the cerebellar cortex^4^. Each longitudinally-striped compartment has a specific molecular expression profile in its PCs, such as zebrin-positive and -negative^5^. By referring to the molecular expression pattern in the cerebellar cortex, the longitudinal compartmentalization (or modular organization) and the topographic olivocerebellar projection pattern have been well clarified in rats and mice^6–9^.

PCs are generated between embryonic day (E)10.5 and E13.5 in mice^10,11^. PCs generated in different time ranges are organized into distinct clusters in the embryonic cerebellum^12,13^. Embryonic PC clusters differentiate into adult longitudinally striped compartments^14,15^, each of which has a particular range of neurogenic timing of PCs^10,11,17^.

IO neurons are generated in the caudal rhombic lip in the period overlapping with that of the PC generation^18–20^. They then migrate tangentially to settle in the ipsilateral ventral medulla^20–22^. Early-generated neurons settle in the dorsolateral parts while late- generated neurons settle in the medioventral parts, producing an area-dependent difference in the neurogenic timing of IO neurons^19,23^. During their migration, their growing axon extends ahead and leads the neuronal migration path. The axon crosses the midline in the ventral medulla, enters the cerebellum through the contralateral cerebellar peduncle, and forms topographic projection to PCs in the embryonic cerebellum^23–25^.

It has been suggested that cell adhesion molecules including cadherin and protocadherin family^26^, and ephrin and ephrin receptors^27^ are involved in the formation of the topographic olivocerebellar projection. However, whether any upstream mechanisms control the expression of cell adhesion molecules in PCs and IO neurons coherently is not known.

Neuronal generation timing is one of the candidates for such upstream factors that may control the expression of cell adhesion molecules to form topographic projection patterns^28^. Early-generated and late-generated neuronal populations project to early-generated and late-generated target populations, respectively, in the output neuronal projection from the accessory and main olfactory bulbs to the telencephalon^29^ and hippocampal dentate gyrus granule cell projection to CA3 pyramidal neurons^30^. However, the relationship between neurogenic timing and the olivocerebellar projection topography still needs to be clarified.

In this study, we systematically examined the neurogenic timing of IO neurons by using the *neurogenin* 2-CreER neurogenic-tagging system^31^. First, IO subdivisions were identified by labeling FoxP2, a specific marker of the IO neurons^16,20^. IO neurogenic timing was then systematically compared with the PC neurogenic timing^11^ of the zebrin stripes of the mouse cerebellum by tracing topographic olivocerebellar projection patterns. The results indicated an inverse relationship between the neurogenic-timing gradients of IO neurons and PCs in the olivocerebellar projection topography. It is the first time this new relationship has ever been reported.

## Results

### Neurogenic timing-dependent reporter labeling in the G2A::H2B mouse IO

The *neurogenin 2* gene is transiently expressed in PCs^32^ and many other neurons, at the transition from a neural progenitor to a differentiating neuron, or neurogenesis. The G2A (*neurogenin 2*-CreER) mouse line is designed to label neurons which are generated at the tamoxifen administration timing^28,33^. In the ventral surface of the medulla of the whole-mount preparation of G2A::Tau^mGFP-nLacZ^ mice, different parts of IO appear to be labeled dependent on the timing of tamoxifen administration (Supplementary Fig. 1), indicating they are composed of neurons of different neurogenic timing.

**Figure 1:**
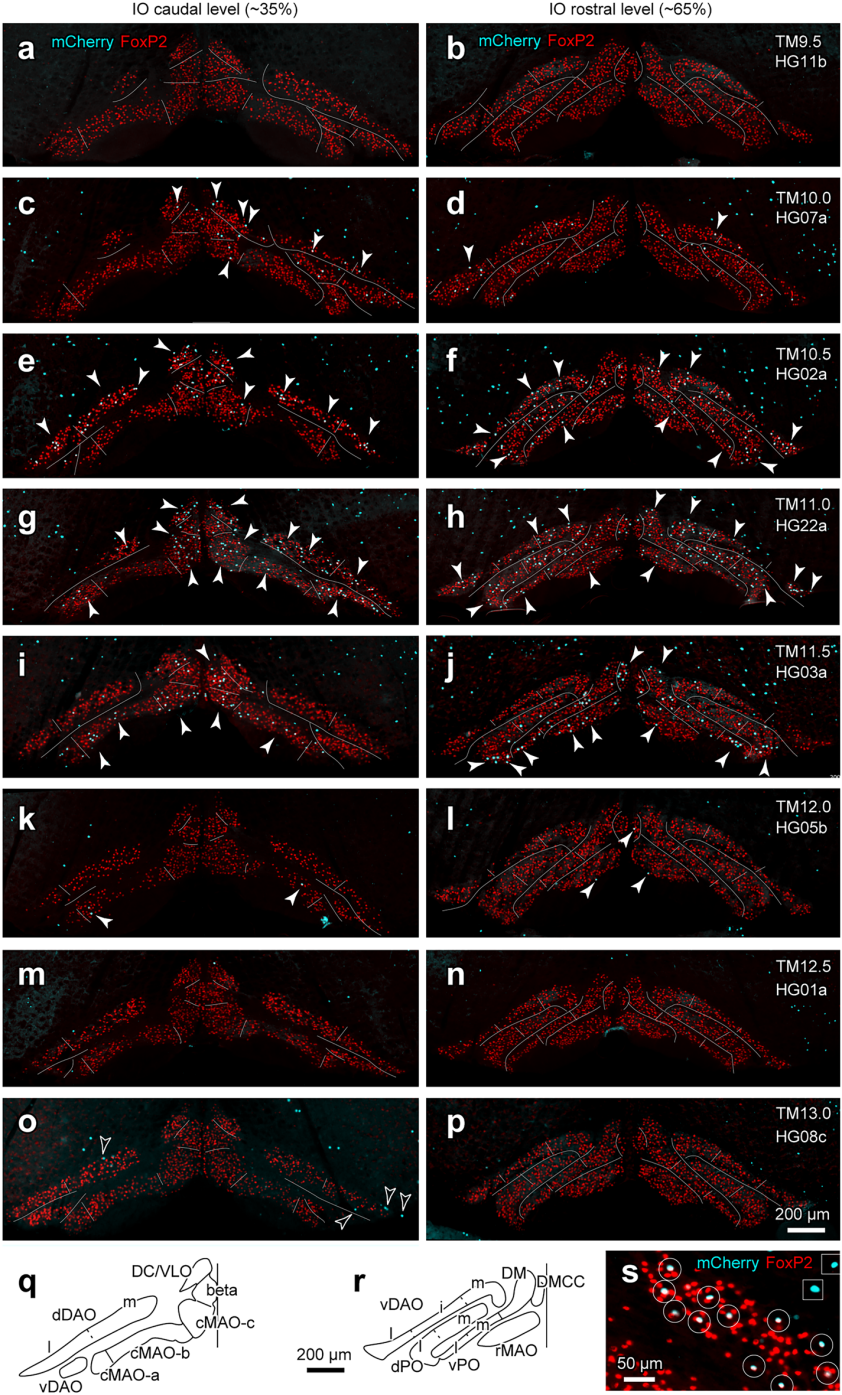
Neurogenic timing-dependent H2B reporter labeling of IO neurons. (**a**–**p**) Coronal sections of the IO at approximately 35% (left panel) and 65% (right panel) levels from the caudal edge in eight G2A::H2B mice with tamoxifen injection at different embryonic stages (TM9.5, 10.0, 10.5, 11.0, 11.5, 12.0, 12.5, and 13.0). Signals indicate immunostaining for FoxP2 (red) and H2B reporter labeling (cyan). Subdivisions of the IO are indicated by white lines. Filled and open arrowheads indicate some reporter-labeled neurons with and without FoxP2 labeling, respectively. (**q**, **r**) Definition of IO subdivisions shown in drawings of left IO in coronal sections at the two levels. “i”, “l” and “m” indicate the intermediate, lateral and medial subdivisions of some IO subnuclei. (**s**) Higher magnification of the IO in G2A::H2B mouse with TM10.5. H2B reporter labeling in IO neurons was co-localized with the FoxP2 labeling (white circles). On the contrary, H2B reporter labeling outside the IO was not co-localized with the FoxP2 signal (white squares). The imaged area of (**s**) is shown in Supplementary Fig. 2 with a square. Created with Adobe Illustrator-10.3, Adobe Photoshop-7.0, and Zen 2.6. See the separate list for abbreviations.

Initially, we looked at the labeling of IO neurons in G2A::Ai9::AldocV mice at about postnatal day (P) 40 with tamoxifen injection at embryonic stages^11^. Although IO neurons appeared to be labeled in these mice, dense labeling of presumable dendritic arbors prevented reliable counting of the number of labeled IO neurons (Supplementary Fig. 2a). Therefore, we used another reporter mouse strain, R26R-H2B-mCherry (abbreviated as “H2B”), which expresses fluorescent protein mCherry fused with the nuclear localization sequence^34^. In G2A::H2B mice with tamoxifen injection, the mCherry signal that is indicative of neurogenic timing-dependent labeling (designated as “(H2B) reporter labeling” or “reporter-labeled” in this report) was localized only in the nucleus with all-or-none type high contrast (Fig. 1s, Supplementary Fig. 2b, d), which facilitated quantitative observation.

To compare the efficiency of reporter labeling between Ai9 and H2B mice, we looked at zebrin stripes lateral 3− and 4+ in the simple lobule in G2A::Ai9::AldocV and G2A::H2B::AldocV mice that were given tamoxifen at E10.5, which is the timing of PC generation in these stripes^11^. The number of labeled PCs was smaller in G2A::H2B::AldocV mice than in G2A::Ai9::AldocV mice (Supplementary Fig. 2c, d). This indicated that the labeling efficiency of H2B reporter mice was lower than that of Ai9 mice (see “Discussion”), although they both reported consistent labeling as a Cre reporter and possessed the normal zebrin striped pattern in the cerebellum (Supplementary Fig. 2e–g).

### Definition of IO subdivisions with FoxP2 immunostaining

Immunostaining of FoxP2 labels almost all neurons in the IO but scarcely labels other nearby neurons^16^. The FoxP2 immunostaining signal was located in the nucleus of neurons, facilitating clear recognition of neuronal distribution (Supplementary Fig. 3). Consequently, we could count the number of IO neurons readily and recognize the boundaries of IO subdivisions in preparations with FoxP2 immunostaining. We adopted the standard subdivision nomenclature and boundary definition of the rat and mouse IO^4,35^. Furthermore, we redefined subdivision boundaries in several places where the boundaries have not been clearly defined.

The IO is composed of five major lamellas which are generally well separated from one another; the dorsal and ventral folds of the dorsal accessory olive (dDAO, vDAO), dorsal and ventral lamellas of the principal olive (dPO, vPO), and medial accessory olive (MAO). In some particular areas, where lamellas are merged to make their distinction difficult, we temporarily defined the boundary at the position of sparse neuronal distribution as follows. The rostrolateral edge of the IO, where the vDAO and the dPO tend to merge, was included in the vDAO (Supplementary Figs. 3i, 4j), similar to the study with glutamate decarboxylase immunostaining in the rat IO^35^. The “bend” of the principal olive (PO), where the dPO and vPO merge laterally^4^, was regarded as a part of the dPO because a gap of neurons was sometimes observed between the bend and the main part of the vPO (Supplementary Figs. 3f, g, 4f–h). The medial part of the vPO is continuous with the dorsomedial subnucleus (DM), although the DM and the vPO have different topographic projection patterns^7,9^. We defined the boundary between the DM and the vPO at the position under the bulb-shaped medial end of the vDAO since neuronal density was often low at this position (Supplementary Figs. 3g–i, 4f–i). The medial accessory olive is separated into the caudal and rostral parts (cMAO and rMAO) that have different topographic projections. The boundary between the cMAO and rMAO was defined at about the 55% level in the caudorostral range of the IO, where the density of neuronal distribution was low. In addition, the rMAO defined as above showed much higher immunoreactivity to protocadherin 10 (Pcdh10, Supplementary Fig. 3f–i) than any part of the cMAO, supporting this definition.

The cMAO is divided into the lateral, intermediate, and medioventral parts that have been named subnuclei a, b and c of the cMAO (cMAO-a, cMAO-b, and cMAO-c^36^), respectively, which project to different zebrin stripes in the cerebellar cortex^7,9^. Although these subareas are not necessarily clearly distinguished from one another, we often saw a gap of neurons in the consistent lateral part of the cMAO (Open arrowheads in Supplementary Fig. 3a–c). We tentatively defined this gap as the boundary between the cMAO-a and cMAO-b in the present study. The cMAO-c expresses the *pcdh10* gene as reported in a study with *pcdh10*^lacZ/+^ mice^26^. Pcdh10 immunostaining in the present study showed moderate immunoreactivity in the cMAO-c (filled arrowheads in Supplementary Fig. 3a–e). We tentatively defined the gap of neurons observed in the medialal edge of the Pcdh10 immunoreactive area as the boundary between the cMAO-b and cMAO-c.

The cMAO-c, the subnucleus beta, and the dorsal cap (DC) or ventrolateral outgrowth (VLO) subnucleus form the vertically-column-shaped medial part in the caudal IO. In this column-shaped part, boundaries were generally recognized by the partial gap of neuronal distribution. Furthermore, the DC showed moderate Pcdh10 immunoreactivity whereas the beta did not. The boundary between the beta and the DM was recognized at about 55% level in which neuronal density was low at their junction.

In serial sections of the IO with FoxP2 immunostaining, the number of FoxP2-positive IO neurons was counted in each IO subdivision (Table 1). The total number of IO neurons obtained in the present study (25,470 neurons, Table 1) was comparable to the results of the preceding study (12,610 neurons on one side of the mouse IO^37^), supporting that FoxP2 is expressed in almost all IO neurons.

**Table 1:** Number of neurons in the mouse IO subdivisions.

Subdivisions	cMAO-a	cMAO-b	cMAO-c	beta	DC/VLO	dDAO	vDAO	dPO	vPO	DM	DMCC	rMAO	Total
Left	582	1852	1003	701	472	645	2525	1433	1054	1011	115	1328	12,721
Right	749	1876	1381	694	383	604	2445	1183	1028	990	133	1283	12,749
Total	1331	3728	2384	1395	855	1249	4970	2616	2082	2001	248	2611	25,470

Counted in serial sections of a G2A::H2B mouse immunostained for FoxP2.

In addition to defining subdivisions of the IO above, we arbitrarily divided the four large subdivisions into several parts to count the number of labeled neurons separately. The vDAO was divided into three parts of approximately equal widths (medial, intermediate, and lateral parts) in the mediolateral direction, whereas the dDAO, dPO, and vPO were divided into the medial and lateral parts of approximately equal widths (Fig. 1q, r, Supplementary Fig. 4d–j).

### Neurogenic timing variation among IO subdivisions

We observed preparations of 20 G2A::H2B mice injected with tamoxifen at E9.5 (n = 1), E10.0 (n = 3), E10.5 (n = 3), E11.0 (n = 3), E11.5 (n = 4), E12.0 (n = 3), E12.5 (n = 1), E13.0 (n = 1), and E13.5 (n = 1). Reporter labeling was almost absent in the IO with tamoxifen injection at E9.5 (“TM9.5”, Fig. 1a, b). It emerged sparsely in the dDAO, vDAO, dPO, DC/VLO, and beta with TM10.0 (Fig. 1c, d), and increased in these subdivisions and emerged sparsely in all other IO subdivisions with TM10.5 (Fig. 1e, f). It further increased generally with TM11.0 (Fig. 1g, h), nearly disappeared in the dDAO and vDAO but remained in other subareas with TM11.5 (Fig. 1i, j). It almost disappeared except for the very sparse labeling in the cMAO, rMAO, and DMCC with TM12.0 (Fig. 1k, l), and disappeared completely in the IO with TM12.5 or TM13.0 (Fig. 1m–p). It was observed only in FoxP2-negative neurons with TM12.5 or TM13.0 (open arrowheads in Fig. 1o). Although we did not characterize these lately-generated neurons, they may belong to the population different from the climbing fiber-projecting population^38^.

**Figure 2:**
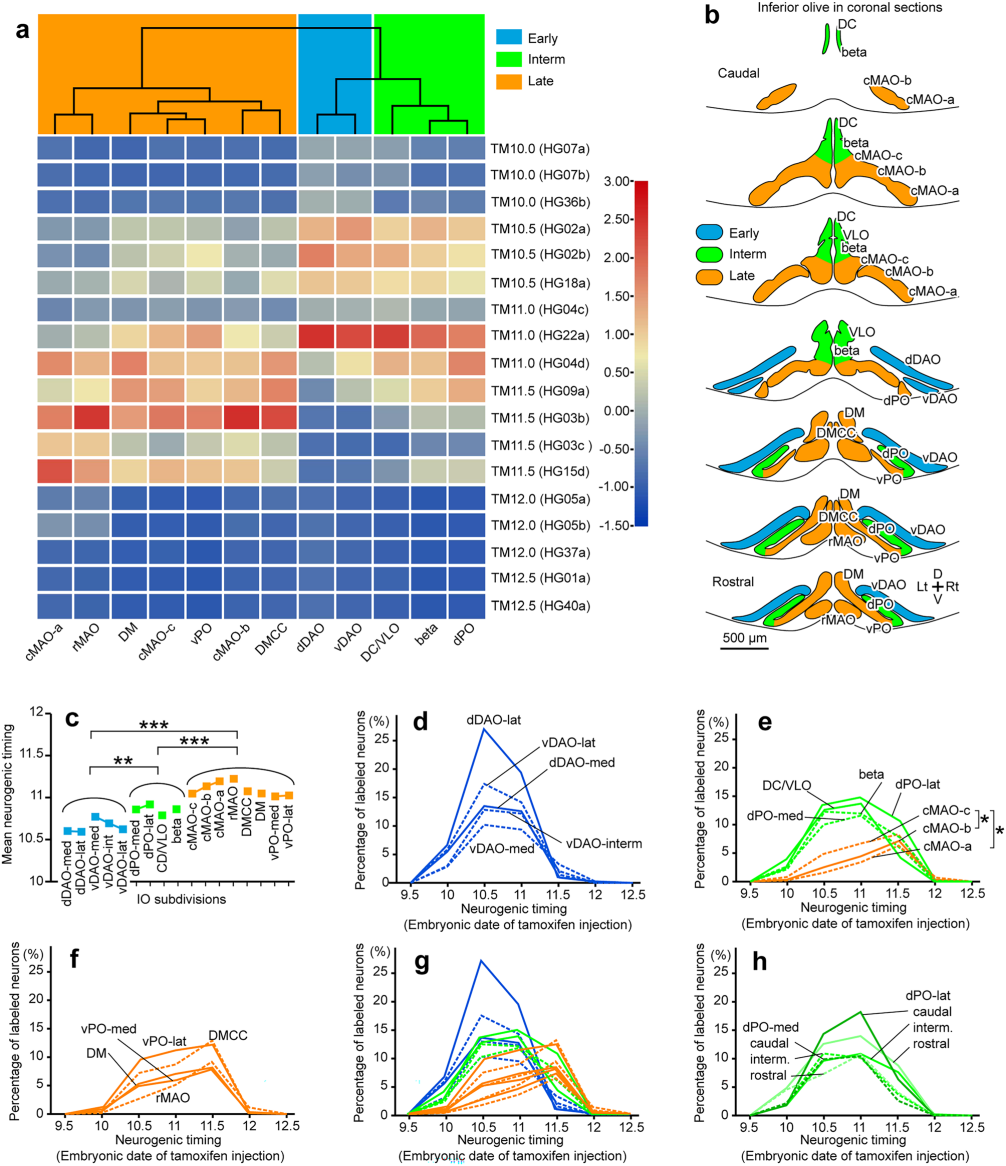
Neurogenic timing distribution of neurons in different subdivisions of the IO. (**a**) Classification of IO subdivisions into early (blue), intermediate (green) and late (orange) groups based on the unbiased clusterogram. The clustrogram was obtained from the matrix table of the number of labeled neurons divided by the total number of neurons in each mouse given tamoxifen at various embryonic date (rows), and for each major IO subarea (columns; original data in Supplemementary Table 2). The top three groups (orange, blue and green areas of the clusterogram) showed apparently different tamoxifen-date-dependent labeling patterns. Consequently, they were defined as late (orange), intermediate (green) and early (blue) groups. Color bar in the right shows the color coding of the value, subtracted by the average and then divided by the standard deviation, of each cells in the matrix table (between 3.0 and − 1.5). (**b**) Classification of IO subdivisions into the three groups defined in (**a**) shown on schematic drawing of the IO in coronal sections at caudorostral levels of 10%, 19%, 26%, 35%, 45%, 61%, and 71% (from top to bottom) from the caudal edge. (**c**) Mean neurogenic timing obtained for all (small) subdivisions classified into the three groups defined in (**a**). Symbols connected by a line indicate mediolateral subdivisions of a IO subnucleus. Mean neurogenic timing was significantly different among early (blue), intermediate (green) and late (orange) groups and between all pairs of groups (one-way ANOVA, P < 0.0001, F(2,14) = 56.50, and post-hoc Bonferroni test, adjusted P = 0.0034 between early and intermediate, adjusted P < 0.0001 between early and late, adjusted P = 0.0003 between intermediate and late). (**d**–**f**) Change of the percentage of the neurons labeled with the neurogenic-tagging system with different tamoxifen injection timing (abscissa) in smaller IO subdivisions: dDAO subdivisions (-lat, -med) and vDAO subdivisions (-lat, -med) (**d**), dPO subdivisions (-lat, -med), cMAO subdivisions (-a, -b and -c) and nearby subdivisions (**e**), vPO subdivisions (-lat, -med), DMCC and rMAO (**f**). The percentage was obtained from 2–4 cases (except for 1 case of TM9.5) of the same tamoxifen injection timing (19 cases in total). Differences in the percentage of labeled IO neurons were tested with two-way ANOVA with the repeated measurement between medial, (intermediate) and lateral subdivisions of major IO subnuclei. Significant difference was observed only between cMAOa and cMAOc (P = 0.042, d.f. = 20, F value = 4.72), and between cMAOa and cMAOc (P = 0.015, d.f. = 20, F value = 7.04). (**g**) Superimposition of all plots in (**a**–**d**). (**h**) Similar analysis for the rostral, intermediate, and caudal temporary subareas of the dPO subdivisions done in 14 cases (two cases of each injection timing). The colors of the graph indicate the early (blue), intermediate (green), and late (orange) groups classified after the analysis. Data were obtained from one TM9.5, three TM10.0, three TM10.5, three TM11.0, four TM11.5, three TM12.0, and two TM12.5 G2A::H2B mice. TM13.0 or TM13.5 mice showed no labeled neurons in any subdivisions (not plotted in the graph). Created with RStudio-2022.12.0.-353, R-4.2.2, pheatmap-1.0.12 for the tree part of (**a**), TBtools-1.112 for matrix part of (**a**), and Adobe Illustrator-10.3. See the separate list for abbreviations.

To quantitatively analyze the neurogenic timing, we counted the reporter-labeled neurons in each IO subdivision on the left and right sides in serial sections (Supplementary Table 2). To classify IO subdivisions based on the timing of reporter labeling in an unbiased way, we applied the hierarchical clusterogram analysis to the matrix data of reporter labeling percentage in every major IO subdivision in all mice of tamoxifen injection at various timing (Fig. 2a). In the obtained clusterogram tree, the top three groups (orange, blue and green areas of the clusterogram) showed apparently different tamoxifen-date-dependent labeling patterns. The major IO subdivisions in the left 7 columns (rMAO, cMAO-a, -b, -c, vPO, DM, and DMCC) had high percentages at TM11.0 and TM11.5 (orange, late group). Those in the right three columns (dPO, beta, and DC/VLO) had high percentages with TM10.5, TM11.0, and TM11.5 (green, intermediate group). Those in the remaining central two columns (dDAO and vDAO) had high percentages with TM10.5 and TM11.0, and low but detectable percentages with TM10.0 (blue, early group).

To confirm the group classification, we measured the mean neurogenic timing from the labeling percentage data for all subdivisions (Fig. 2c). All subdivisions in the early (blue), intermediate (green), and late (orange) groups had the mean neurogenic timing of E10.58–E10.77, E10.78–E10.92, and E11. 0–E11.22, respectively (Fig. 2c), supporting the classification. The distributions of mean neurogenic timings of subdivisions were significantly different among the three groups (Fig. 2c).

**Figure 3:**
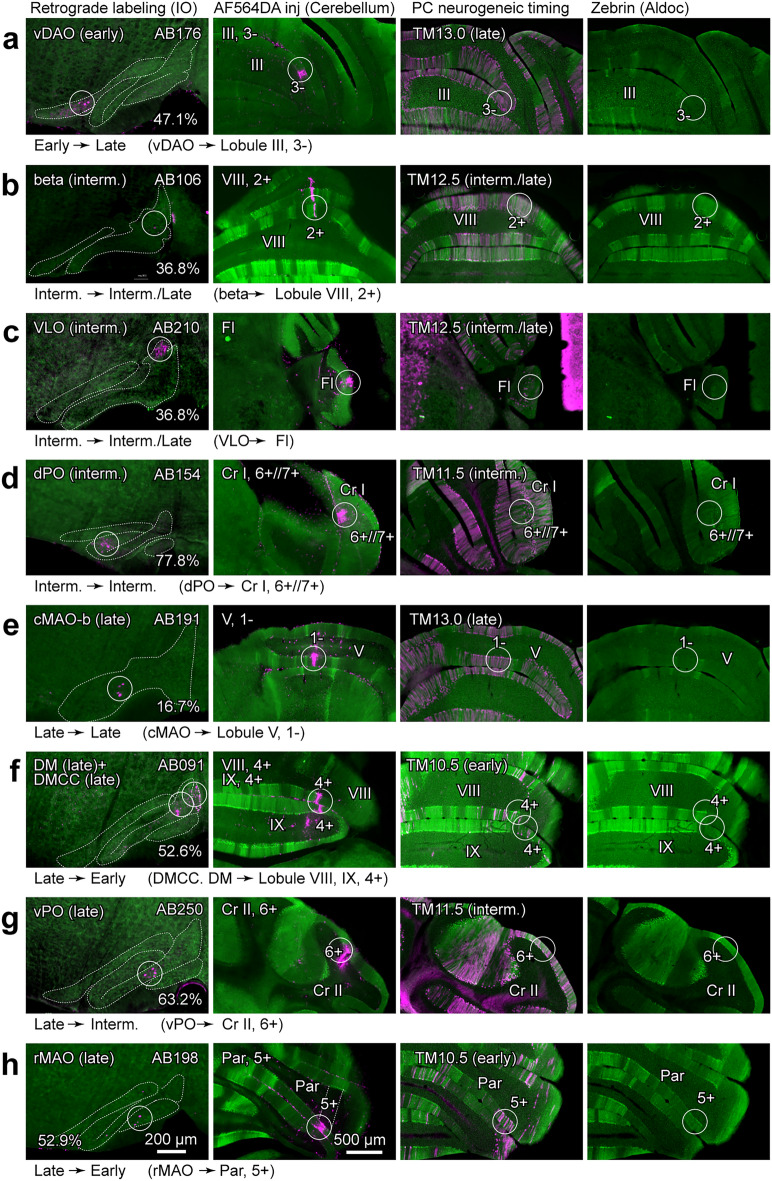
Retrograde tracing of the olivocerebellar topographic projection and consequent identification of the neurogenic timing relationship between the origin (IO) and target (PCs). Distribution of retrogradely-labeled IO neurons in a particular IO subarea (most left panel) labeled by localized injection of AF546DA into a particular area in the cerebellar cortex (second panel from the left), PC labeling by the neurogenic-tagging system in the same area of the cerebellar cortex (second panel from the right) and zebrin pattern of that area (most right panel) are shown for eight different positions (**a**–**h**). Images of Purkinje cell labeling with the neurogenic-tagging system were selected from the data set produced in our previous study performed with G2A::Ai9::AldocV mice and tamoxifen injection at a particular timing of embryonic stage^11^. Images in the second panel from the right and the most right panel show the same section. Circles indicate the distribution of retrogradely labeled IO neurons (most left panel), the AF564DA injection sites (second panel from the left), and approximately the same position as the AF564DA injection sites (second panel from the right and most right panel). The green pseudo-color shows the Venus signal indicating the aldolase C expression in all panels. The magenta pseudo-color shows AF564DA signal in the most left panel and the second panel from the left, and labeling with the neurogenic-tagging system in the second panel from the right. In the second panel from the right in (**c**), dense neuronal labeling are seen in the cerebral cortex and medial brain stem besides labeling in some PCs. Perentage in the most left panel indicates the relative position of the section within the caudorostral extent of the IO (0%: caudal edge, 100%, rostral edge). Scale bars in (**h**) apply to all panels in the most left column (200 µm), and to all panels in the three other columns (500 µm). Created with Adobe Illustrator-10.3, Adobe Photoshop-7.0, and Zen 2.6. See the separate list for abbreviations.

The time course of tamoxifen-dependent labeling was further examined by plotting the percentage of reporter-labeled neurons, averaged from all cases of tamoxifen injections at the same timing, for mediolateral smaller subdivisions of major IO subnuclei (Fig. 2d–f). In all subdivisions of the dDAO and vDAO, labeling started with TM10.0, occurred mostly with TM10.5 and TM11.0, and disappeared with TM11.5 (Fig. 2d). In the DC/VLO, beta, and all subdivisions of the dPO, labeling started with TM10.0, occurred mainly with TM10.5 and TM11.5, but persisted with TM11.5 only to some extent (Fig. 2e). In all subdivisions of the cMAO, rMAO, and all subdivisions of the vPO, the labeling started with TM10.5, remained with TM11.0 and peaked with TM11.5, and nearly disappeared with TM12.0 (Fig. 2e, f). These plots of the percentage of reporter-labeled neurons (Fig. 2d–f) showed different time course patterns of neuronal generation among IO subdivisions as classified by the clusterogram analysis: (1) labeling started at E10.0, peaked at E10.5 and E11.0 and nearly finished by E11.5 (early group, blue lines in Fig. 2d, g), (2) labeling started at E10.0, peaked at E10.5 and E11.0, persisted at E11.5 and finished by E12.0 (intermediate group, green lines in Fig. 2e, g), and (3) labeling started at E10.0, increased at E10.5 and E11.0, peaked at E11.5 and nearly finished by E12.0 (late group, orange lines in Fig. 2e–g). The intermediate group had the longest span of neuronal generation (E10.0–E11.5) among the three groups.

Among mediolateral subdivisions of major subnuclei, the plots of percentage did not show noticeable differences (Fig. 2d–f). However, the mean neurogenic timing was later in the lateral part than in the medial part in the cMAO, and dPO, whereas it was earlier in the lateral part than in the medial part in the vDAO (plots connected with lines in Fig. 2c). Further analysis with the percentage labeling data showed that this difference was significant only between cMAO-b and cMAO-c (P = 0.042), and between cMAO-a and cMAO-c (P = 0.015). In the dPO, which belonged to the intermediate group, we additionally defined three rostrocaudal temporary subareas and re-analyzed the number of reporter-labeled neurons. No clear difference was observed among the rostrocaudal subareas (Fig. 2h).

The results, as a whole, indicated that the order of neurogenic timing was essentially arranged in accordance with the dorsolateral-ventromedial lamellar organization. The dorsolateral lamellas, i.e., the dDAO and vDAO, contained mainly early-generated neurons; the intermediate lamellas, i.e., the DC/VLO, beta, and dPO, contained both early-generated and late-generated neurons; whereas the ventromedial lamellas, i.e., DM, vPO, DMCC, cMAO, and rMAO contained more late-generated neurons than early-generated neurons (Fig. 2b). The cMAO had a mediolateral gradient of the neurogenic timing inside the single lamella. The results here generally matched with the neuronal labeling pattern observed in the whole brain preparation (Supplementary Fig. 1).

### Relationship between the neurogenic timing and the topography of the olivocerebellar projection

To identify the topographic olivocerebellar projection pattern in various IO subdivisions, we labeled IO neurons retrogradely (Fig. 3, the most left panel) by injecting neuronal tracer AF546DA into various compartments of the cerebellar cortex in Aldoc-Venus mice (Fig. 3, the second panel from the left). Then, we looked at the data set of IO neurogenic timing in IO subdivisions (Fig. 2b) as well as the data set of Purkinje cell neurogenic timing in our previous study with the neurogenic-tagging system^11^ (Fig. 3, the second panel from the right, and most right panel). The neurogenic timing range of PCs in cortical compartments was classified into “early” (high degree labeling with TM10.0–TM12.0), “intermediate” (TM10.5–TM12.5), “late” (TM12.5–TM13.0) and “latest” (TM12.5–TM13.5) in the present analysis.

This analysis indicated that a part of the vDAO, in which IO neurons were generated in the early timing, projected to stripe 3- in lobule III, in which PCs were generated mainly in the late timing (Fig. 3a). Parts in the beta subnucleus, VLO and dPO, in which IO neurons were generated in the intermediate timing, projected to stripe 2+ in lobule VIII, flocculus, and stripe 6+//7+ in crus I, respectively. In these cortical areas, PCs are mainly generated at the intermediate timing (Fig. 3d) or intermediate and late timing (Fig. 3b,c). Parts in the cMAO, DMCC + DM, vPO, and rMAO, in which IO neurons were generated in the late timing, projected to stripe 1− in lobule V, stripe 4+ in lobules VIII and IX, stripe 6+ in crus II, and stripe 5+ in the paramedian lobule. In these cortical areas, Purkinje cells are generated in the late (Fig. 3e, h), early (Fig. 3f), and intermediate (Fig. 3g) timings. The results indicated that the neurogenic timing of IO neurons was not simply related to the neurogenic timing of innervated PCs. On the contrary, there was often, but not always, a reverse relationship between them, i.e. early- and late-generated IO neurons often innervated late- and early-generated PCs, respectively.

**Figure 4:**
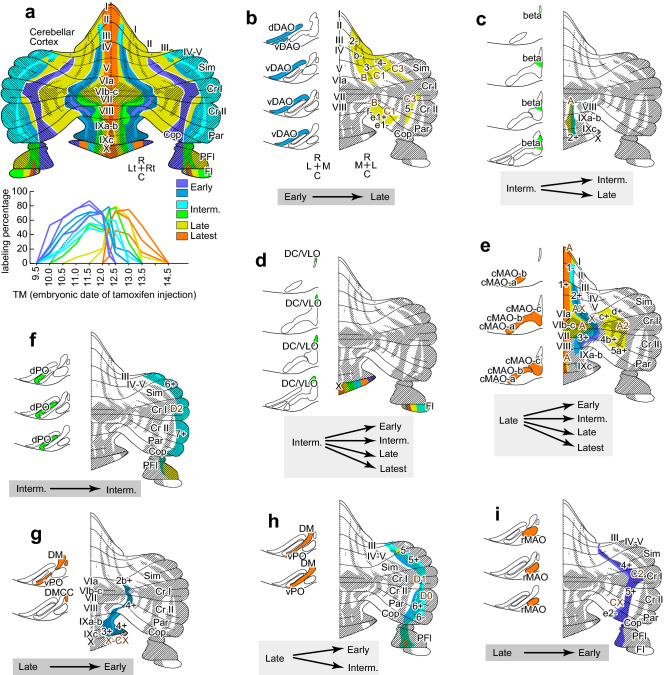
Summary diagram of the neurogenic timing relationships in the modular subsets of the olivocerebellar projection. (**a**) Neurogenic timing of PCs in the cerebellar cortex. Mapping of distributions of PCs of different neurogenic timing (top) and graphs of neurogenic period for each distribution area (bottom), based on Zhang et al.^11^ with permission. (**b**–**i**) Relationship of neurogenic timing of IO subdivisions and compartments in the cerebellar cortex that are topographically connected by the olivocerebellar projection. The IO subdivision that is focused on is colored on the left side in each panel. The cortical compartments that are topographically projected by the IO subdivision are colored on the right side. The relationship of neurogenic timing between the IO and PCs is indicated at the bottom of each panel, in which dark gray shadowing indicates the major relationship. In this figure, the unfolded representation of the cerebellar cortex with the longitudinally-striped zebrin (aldolase C) expression pattern is based on previous studies^5–7,48^. Shadowed and non-shadowed areas represent aldolase C-positive and -negative stripes, respectively. The topographic relationship of the olivocerebellar projection pattern is based on previous reports in mice^9^ and rats^6–8,39,47^. The neurogenic timing of cerebellar PCs in each compartment or module is based on Zhang et al.^11^. The neurogenic timing of neurons in each IO subdivision is based on the results of the present study. See the separate list for abbreviations. Created with Adobe Illustrator-10.3.

To further examine this relationship, we mapped the reported topographical relationship of the olivocerebellar projection in mice and rats^6–9,39^ on the reported neurogenic timing of striped subareas of the cerebellar cortex in the mouse (Fig. 4a)^11^. The dDAO and vDAO (early IO subdivisions, blue in Fig. 4b) project to the zebrin-negative and zebrin-faintly negative stripes in the paravermis and hemisphere, which corresponds to modules B and C1/C3^6–9,39^. These zebrin stripes belong to late compartments (yellow in Fig. 4b)^11^. The beta subnucleus (intermediate IO subdivisions, green in Fig. 4c) projects to stripe 2 + in lobule VIII–IX, which contains intermediate (lateral stripe 2 +) and late (medial stripe 2 +) compartments (green and yellow, respectively, in Fig. 4c). The DC/VLO (intermediate IO subdivisions, green in Fig. 4d left) projects to the flocculus and nodulus (lobule X), which contains early, intermediate, late and latest compartments (various colors in Fig. 4d right). The dPO (intermediate IO subdivisions, green in Fig. 4f left) projects to the most lateral area in the hemisphere (module D2), which contains mostly intermediate compartments (cyan, Fig. 4f right). The cMAO-a, cMAO-b, and cMAO-c (late IO subdivisions, orange in Fig. 4e) project to most of the vermal areas (modules A, AX, and X) and paravermal areas in the simple lobule, crus I, crus II and paramedian lobule (module A2), which contain early, intermediate, late and latest compartments (various colors in Fig. 4e right). The DMCC and the most caudal parts of the DM and vPO (late IO subdivisions, orange in Fig. 4g left) project to the areas in the lateral vermis (module X-CX) which contains the early compartment (blue in Fig. 4g right). The rest of the vPO and the DM (late IO subdivisions, orange in Fig. 4h left) project to the stripes in the medial hemisphere (Fig. 4h right), which contains early and intermediate compartments. The rMAO and the caudal end of the cMAO-b near the rMAO (late IO subdivisions, orange in Fig. 4i left) project to the zebrin-positive and negative stripes in the lateral paravermis (modules C2 and CX), which contains early compartments (purple in Fig. 4i right).

The topographic olivocerebellar projection pattern we demonstrated above (Fig. 3) fully agreed with the pattern reported in previous studies (Fig. 4)^6–9,39^. Based on the topographic projection pattern, the relationship between the neurogenic timing ranges of the origin (IO subdivisions) and target (cerebellar cortical compartments of PCs) was clarified for the entire olivocerebellar projection. Although the relationship was variable among subdivisions, early-, intermediate-, and late-neurogenic IO subdivisions often projected to the late-, intermediate-, and early-neurogenic PC compartments (highlighted at the bottom of Fig. 4b, f, g, i). The result indicates that the topography in the majority of the olivocerebellar projections is formed according to the reverse neurogenic-timing gradients between IO subdivisions and the cerebellar compartments.

## Discussion

**Figure 5:**
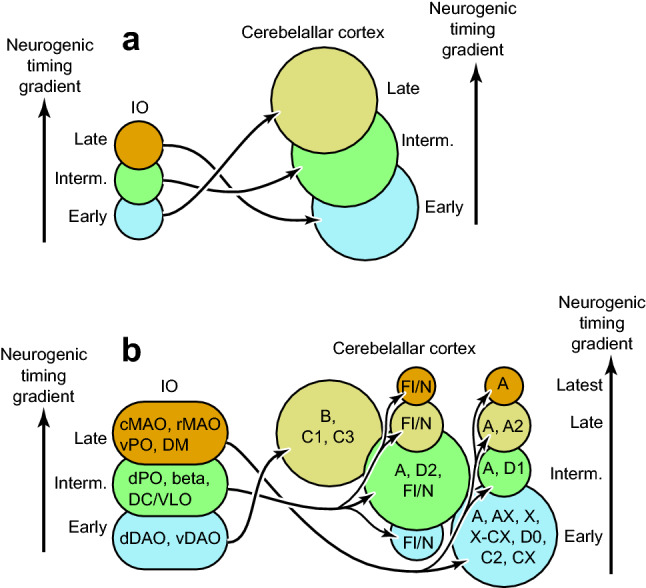
Speculation on the formation of the olivocerebellar topographic connection in relation to the neurogenic timing gradient. (**a**) The basic topographic olivocerebellar projection pattern in relation to the neurogenic timing gradients of IO neurons and PCs in the cerebellar cortex. Here, the topographic connection is formed in the way opposite to the neurogenic timing order; the IO subdivisions that contain early-, intermediately-, and late-generated neurons project to the cortical compartment that contains late-, intermediately-, and early-generated PCs, respectively. (**b**) The secondary neurogenic timing-dependent subdivisions of the cerebellar cortex are additionally formed in particular cortical areas. These secondary subdivisions receive projections from the nearby IO subdivisions of the same neurogenic timing (vertical spread of arrows). This scheme can accommodate the mouse olivocerebellar projection pattern between the entire IO and entire cerebellar cortex. IO subdivisions and cortical modules that belong to each element are indicated. See the separate list for abbreviations.

In the present study, we clarified the neurogenic timing of IO subdivisions and re-examined the topographic relationship between IO subdivisions and the cortical compartments in the olivocerebellar projection. Referring to the neurogenic timing of PCs in the cerebellar cortex, we have shown the general reverse relationship in the neurogenic-timing gradient in the olivocerebellar projection (Fig. 5). The developmental relevance and functional significance of the findings are discussed here.

### Neurogenic timing-specific labeling of IO neurons

Neurogenic timing of IO neurons has been studied with the ^3^H-thymidine^40^ and 5-bromo-2′-deoxyuridine (BrdU)^19^ labeling methods. The study with BrdU injection at 4-h intervals in the rat^19^ has shown that IO neurons are generated between E11.7 (E11 + 16 h) and E13.3 (E13 + 8 h)^19^. This study also demonstrated that neuronal generation occurs early in the DAO, intermediately in the PO, DC/VLO, and subnucleus beta, and late and with mediolateral gradient in the MAO^19^. The neurogenic-timing difference among IO subdivisions observed in the mouse in the present study was generally similar to the result in the rat study^19^, corroborating the neurogenic timing-specific labeling of murine IO neurons in G2A mice. The neurogenic-tagging system with G2A mice^31^ is a type of genetic inducible fate mapping^41^. While ^3^H-thymidine and BrdU label progenitor cells in the S phase at the time of injection, *Neurog2*, the gene that is manipulated in G2A mice, is expressed transiently in the transition time window from a neural progenitor to a differentiating neuron in many brain areas^31,32^. The peak of Cre-dependent reporter expression occurs 6–12 h after the final DNA synthesis in G2A mice in most neurons^33^. If injected at the same timing, tamoxifen would label neurons generated 6–12 h earlier than BrdU and ^3^H-thymidine.

The H2B reporter mouse used in the present study had apparently an expression efficiency lower than that of the Ai9 reporter mouse (Supplementary Fig. 2). This is presumably related to the length of the STOP cassette between the two loxP sites (about 2.7 kb in H2B^34^, and about 0.9 kb in Ai9)^42^. Nevertheless, the nucleus-localized expression of fluorescence facilitated counting labeled IO neurons in the present study.

### Definition of IO subdivisions

The IO subdivisions are defined primarily by their cytoarchitecture. However, their link to the topographic axonal projection to the cerebellar cortical compartments is also characteristic^6,7,9^. By using immunostaining of FoxP2, which marks climbing fiber-projecting neurons with specificity^16^, we have confirmingly defined most of the boundaries between IO subdivisions to make a quantitative analysis of reporter-labeled neurons. In some parts where we needed to newly define subdivisonal boundaries in this study, we assumed the gap or a low density in FoxP2-positive neuron distribution as a boundary between subdivisions of IO. Accordingly, we have proposed some new definitions of IO boundaries. For example, the most lateral “bend” area of the PO has been considered to belong to the dPO. Such proposals are to be validated in terms of the axonal projection pattern in the future.

Pcdh10 immunostaining is useful in identifying the rMAO in the present study. Our previous study has shown expression of the *pcdh10* gene in the rMAO as well as in neurons in the lateral part of the posterior interpositus nucleus and in PCs in zebrin-positive stripe 4+//5+^26^. All these three areas are topographically connected through the olivocerebellar and PC projections^26^. Thus, the expression of homophilic cell-adhesion molecule Pcdh10 in the rMAO is rather expectable. However, the Pcdh10 immunostaining signal in the DC or cMAO-C, also expected because of the *pcdh10* gene expression^26^, is less strong than in the rMAO.

Cytoarchitectonic studies recognized the lateral and medial arcuate nuclei, special small subdivisions located ventrolateral to the vPO and ventral to the lateral edge of the rMAO, respectively, in the IO of the C57BL/6 mouse strain^43,44^, to which mice of the present study belong. The lateral and medial arcuate nuclei are speculated to be a part of the vPO. Whereas we recognized the lateral arcuate nucleus and included it in the vPO (c.f. Supplementary Figs. 3h, 4j), almost all IO neurons in the rMAO, except for some scattered neurons in the ventral edge (c.f. Supplementary Fig. 3g, h) showed Pcdh10 immunoreactivity surrounding the FoxP2 immunoreactivity. Thus, we could not clearly confirm the medial arcuate nucleus which is supposed to be negative in Pcdh10 expression if it belongs to the vPO.

### Possible mechanisms for the relationship between neurogenic timing and olivocerebellar topography

The IO is a well-distinguished structure composed of neurons that give rise to climbing fibers, although a small number of different types of neurons, such as GABAergic neurons, are present inside or near the IO^35,38^. The IO is formed during the period between E13 and P0 in the mouse by the arrival of migrating IO neurons from the lower rhombic lip to the final IO position^20^. Since IO neurons migrate by moving upstream inside the leading axonal path^20^, the topography of the axonal projection and the formation of IO subdivisions seem to be highly linked to each other. How the neurogenic timing of IO neurons is related to these things is the question.

One mechanism to explain the formation of the topographic projection between the origin and target is that their matched neurogenic timing allows newly growing axons to find unoccupied targets in newly growing dendritic arbors^29^. Accordingly, newly generated origin neurons tend to project to newly generated target neurons, as shown in the olfactory bulb projection^29^ and the dentate gyrus granule cell projection^30^. However, the reverse relationship found in the present study in the neurogenic-timing gradients between the origin and the target (Fig. 5) indicates that different mechanisms are involved in the formation of the olivocerebellar topography. In the olivocerebellar projection, axon targeting is guided by various molecules such as homeobox genes and the downstream cell adhesion molecules and attractants/repellents molecules, including Eph/Ephrin molecules and cadherin/protocadherin family molecules^25–27,45,46^. We speculate that the reverse neurogenic-timing gradient relationship can be created if the expression of such molecules is controlled partly independently of the neurogenic timing of IO neurons and in PCs.

The results of the present study seem to propose an additional different mechanism for the formation of the topographic connection. In the medial vermis, medial paravermal area in central lobules (simple lobule, crus I, crus II and paramedian lobule) or module A2, and nodulus (lobule X) and flocculus, multiple cortical compartments of different neurogenic timing are innervated by the single or nearby IO subdivisions that have the same neurogenic timing (vertical diversion of arrows in Fig. 5b). Focal secondary differentiation of cortical compartments in these areas may be able to explain the increase in the number of striped cortical areas that contain PCs of different neurogenic timing but are innervated by nearby IO areas. On the contrary, the reverse neurogenic-timing gradient scheme fits with the majority of the cortical compartments or modules (Fig. 5a and three transversal stem arrows in Fig. 5b).

A different possibility is that the neurogenic timing of the cerebellar nuclei, the second target of the olivocerebellar projection^47^, may be involved in the formation of the topography of the olivocerebellar system. So far, no data have been reported about the neurogenic timing of different subdivisions of the cerebellar nuclei. This question is to be addressed in the future.

## Experimental procedures

### Ethics statements

All experiments were performed in accordance with the Japanese Neuroscience Society and Tokyo Medical and Dental University guidelines for laboratory animal care and use. Experimental protocols were approved by the Animal Care and Use Committee (A2021-065A, A2019-187C3) and Gene Recombination Experiment Safety Committee (G2019-020C5) of Tokyo Medical and Dental University. The study was carried out in compliance with the ARRIVE guidelines.

### Mice

Mice were bred and reared in a 12–12-h light–dark cycled condition in the animal facility of the university with freely available food and water. The following genetically manipulated mouse lines were used in the present study: transgenic C57BL/6N-Tg(Neurogenin2-CreER) strain (G2A, CDB:0512T-1^11,31^), transgenic R26R-H2B-mCherry::C57BL/6 reporter strain (CDB0204K^34^; Abbreviated as “H2B” in this report), knock-in Aldoc-Venus (AldocV) strain^11,48^, transgenic B6.Cg-Gt(ROSA)26Sortm9(CAG-tdTomato)Hze/J reporter strain (“Ai9”, The Jackson Laboratory, https://www.jax.org/strain/007909), and Tau^mGFP-nLacZ^ strain (JAX stock #021162, The Jackson Laboratory).

The G2A strain has a genomic BAC transgene in which the tamoxifen-inducible CreER gene has replaced the coding sequence of the neurogenin 2 (*Neurog2*) gene^31^. This transgene is putatively integrated into the Y chromosome. G2A male mice were mated with C57BL/6N females to obtain the next generation. Genotype was checked in the tail sample by polymerase chain reaction with the primers for the Cre gene (Aki553, TAAAGATATCTCACGTACTGACGGTG, and Aki554, TCTCTGACCAGAGTCATCCTTAGC). Since the CreER is expressed in neuronally committed cells under the *Neurog2* enhancer^31,32^, Cre recombination activity is induced only in the neurons that finish the last mitosis shortly before the tamoxifen administration. G2A males were mated with H2B homozygous females to produce G2A::H2B double-heterozygous hybrid mice, which were mainly used in the present study. We also produced G2A::AldocV(homozygous) hybrid mice. G2A::AldocV males were mated with H2B homozygous females to produce G2A::H2B::AldocV triple-heterozygous mice, which were used in a part of the present study (Supplementary Fig. 2).

The H2B strain is a Cre reporter strain that expresses mCherry fluorescent protein fused with the nucleus translocation sequence^34^. In H2B mice, the genotype was checked in the tail sample by polymerase chain reaction with the primers (P3: TCCCTCGTGATCTGCAACTCCAGTC, P4: AACCCCAGATGACTACCTATCCTCC, and P5: TGTGGAATGTGTGCGAGGCCAGAGG). H2B mice were maintained by mating homozygotes.

The Ai9 strain is a Cre reporter strain that expresses tdTomato fluorescent protein. The AldocV strain expresses Venus fluorescent protein in place of aldolase C protein^48^. Ai9 homozygous females and Ai9::AldocV hybrid homozygous females were mated with G2A males in our previous study to label PCs in the neurogenic timing-specific way^11^. Some preparations obtained in that study were used in parts of Fig. 3 and Supplementary Fig. 2.

Heterozygotes Aldoc-Venus mice, which were used in the neuronal tracing experiments, were produced by mating Aldoc-Venus homozygous males with C57BL/6N females. Tau^mGFP-nLacZ^ strain is a Cre reporter strain that expresses nuclear-targeted beta-galactosidase. Tau^mGFP-nLacZ^ homozygous females were mated with G2A males and the resulting offspring were used as previously described^33^.

### Mating, tamoxifen injection, and harvesting

H2B (or Ai9) homozygous females were mated with G2A (or G2A::AldocV) males. The day when the vaginal plug was detected in mating was designated as E0.5. Tamoxifen (9 mol/l solution in corn oil; T5648-1G, Sigma, St. Louis, MO, U.S.A.) was injected intraperitoneally (2.25 µmole/mouse) one time at noon when the embryos were E9.5, E10.5, E11.5, E12.5, or E13.5, or at midnight when the embryos were E10.0, E11.0, E12.0 or E13.0. This procedure was abbreviated as TM9.5, and so on in the text. This dose is enough effective in labeling neurons in a neurogenic timing-specific manner^11^. The pregnant females were sacrificed by cervical dislocation to take out E19.5 embryos with Cesarean section. Male pups were reared by a stepmother. At postnatal day (P) 40-42, mice were overdosed with an intraperitoneal injection of pentobarbital sodium (0.18 mg/g body weight) and xylazine (0.009 mg/g body weight) or medetomidine hydrochloride (1.5 µg/g body weight), midazolam (8.0 µg/g) and butorphanol tartrate (10.0 µg/g). They were perfused transcardially with phosphate-buffered saline (PBS, pH = 7.4) plus heparin sulfate (0.1%), and then with 4% paraformaldehyde. The brain was dissected after overnight postfixation in 4% paraformaldehyde, and soaked in 30% sucrose with phosphate buffer (pH = 7.4) for two days. Brain samples were stored in the deep freezer before cutting.

### Histological procedures and immunostaining

The ventral medulla was dissected from the brain and coated with gelatin solution (10% gelatin, 10% sucrose in 10 mM phosphate buffer, 32 ºC). The gelatin block was hardened by chilling and then soaked overnight in a fixative with a high sucrose content (4% paraformaldehyde, 30% sucrose in 0.05 M phosphate buffer, pH 7.4). Complete sets of serial sections were cut coronally using a freezing microtome at a thickness of 40 µm. Sections were mounted on glass slides or rendered to immunostaining as follows. After washing in PBS and PBS with 0.12% Triton X-100 (PBST), each complete set of sections was processed for immunostaining. Floating sections were incubated on a shaker with goat anti-FoxP2 (Everest Biotech EB05226, 1:5000) antibody and a choice of the following antibodies, rabbit anti-FoxP2 (Abcam, ab172320, 1:1000, three cases), and rat anti-protocadherin 10 (Millipore, MABT20, 1:1500, six cases) in PBST plus 2% normal donkey serum for 72 h at 4 °C (Supplementary Table 1). The sections were then incubated with a mixture of appropriate secondary antibodies that were conjugated with fluorescent tags (Supplementary Table 1). Finally, these sections were mounted on glass slides, dried, and coverslipped with a water-soluble mounting medium (CC mount, Sigma C9368-30ML).

### Specificity of antibodies

The goat polyclonal anti-FoxP2 antibody (EB05226, Everest Biotech, Oxfordshire, UK) that was consistently used in the present study produces bands at 22, 32, 84, and 144 kDa in the P4 mouse cerebellum, the P4 mouse whole brain, and the adult mouse cerebellum in our hands^49^. Another mouse anti-FoxP2 antibody (Ab172320, Abcam plc, Cambridge, UK), which was used to check specific labeling, labels bands at 51, 67, 75, and 77 kDa in HEK293 whole cell lysate and Hela whole cell lysate according to the Manufacturer’s datasheet. Although the western blot results do not match very well, these two antibodies labeled the same population of neurons in the mouse IO (see “Results”). The rat monoclonal anti-Pcdh10 antibody (MABT20 clone 5G10, Millipore, Billerica, MA) produced a single band of 137 kDa in the P0 mouse cerebellum and P0 mouse whole brain in our western blot^49^. This band disappeared completely in samples from the Pcdh10-KO mouse cerebellum^49^.

### Retrograde labeling of IO neurons

We labeled IO neurons retrogradely by localized injection of Alexa Fluor 546-conjugated dextran amine (AF546DA; D22911, Molecular Probes, Eugene, OR, USA) as described before^26^ in Aldoc-Venus mice, in which the cerebellar zebrin stripes are visualized by intrinsic fluorescent protein expression. Briefly, adult heterozygous Aldoc-Venus mice were anesthetized by intraperitoneal medetomidine hydrochloride (0.75 µg/g body weight), midazolam (4.0 µg/g) and butorphanol tartrate (5.0 µg/g). The mouse was placed on a stereotaxic apparatus with the skull fixed at various nose-down rotation angle. A hole was made in the skull. A drop of AF546DA solution (about 10 nl of 10% solution in saline) was injected in various positions in the cerebellar cortex. After a survival period of 5–7 days, mice were sacrificed to dissect the brain as above. Serial coronal sections of 80-µm thickness were cut and mounted in slides. The slides were coverslipped with PBS temporarily to observe under the microscope.

### Acquisition of digital images

Multiple-channel fluorescence images were digitized into a 12-bit gray scale using a monochrome CCD camera (AxioCam ICm 1, Zeiss, Oberkochen, Germany) attached to a fluorescent microscope (AxioImager.Z2, Zeiss) with an appropriate filter set. Images of the left and right IO were digitized with a 10× objective and tiling function of the software (Zen 2.6 blue edition, Zeiss, https://www.zeiss.co.jp/microscopy/products/microscope-software/zen.html) in all serial coronal sections in G2A::H2B mouse brains with a tamoxifen injection. Images were adjusted concerning contrast and brightness, with no further digital enhancement, and assembled using software (Zen 2.6; Photoshop-7.0 and Illustrator-10.3, Adobe, San Jose, CA, U.S.A., https://www.adobe.com/jp/). Fluorescent signals were shown with pseudo-color in the figures.

### Counting the number of IO neurons

Digital image files of serial sections were opened and placed in the working sheet of Adobe Illustrator. Boundaries of the IO subareas were drawn on the image of FoxP2 immunostaining in all individual sections in Adobe Illustrator (cf. Supplementary Fig. 4). When Pcdh10 immunostaining was performed, the labeling pattern was also referred to in identifying boundaries. The number of FoxP2-positive nuclei was counted manually in each IO subdivision in all serial sections. We did not count labeled objects that had apparently different sizes, shapes or qualities from that of the ordinary nuclei of IO neurons. There was the possibility that the IO neurons that were located at the section-cutting plane were counted in two sections. No correction for this error was made in this study. The percentage of reporter-labeled neurons in each IO subdivision was obtained by dividing the number of reporter-labeled neurons by the number of FoxP2-positive neurons, which was counted in one mouse (HG03b, Table 1).

### Statistical analyses

Statistical analyses (Student’s t-test and analysis of variance, ANOVA with post hoc Bonferroni's multiple comparison test) were done with Excel 2016 (Microsoft) and GraphPad Prism 9.5.1 (https://www.graphpad.com). In Figures, single, double and triple asterisks indicate P < 0.05, < 0.01 and < 0.001, respectively. Unbiased clusterogram analysis to obtain density maps shown in Fig. 2a was performed with pheatmap-1.0.12 software (https://cran.r-project.org/web/packages/pheatmap/index.html) incorporated into the RStudio-2022.12.0.-353 of R-4.2.2 (https://posit.co/download/rstudio-desktop/). The total codes we wrote in RStudio were as follows.
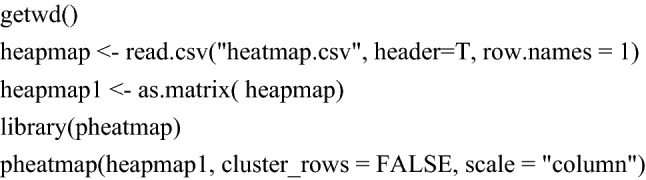


The Heatmap Illustrator function of TBtools-1.112 software^50^ (https://github.com/CJ-Chen/TBtools) also produced the same results. For these analyses, we first prepared an Excel or csv matrix table of the percentage of reporter-labeled IO neurons located in IO subareas for all mice of various tamoxifen injection timing. In case of TBtools, by inputting the matrix table data to TBtools software and selecting the scale method “column scale”, the data in each column (percentage of labeled IO neurons in each IO subarea) were linearly normalized by subtracting the average and then divided by the standard deviation. Then, the algorithm of the software gave the density map which showed the grouping results among different IO subareas.

To compare the neurogenic timing among different IO subdivisions, we introduced “mean neurogenic timing (MNT)” for each IO subdivision. MNT was obtained by:

$${\text{MNT}} = \frac{{9.5{\text{*P}}\left( {{\text{TM}}9.5} \right){ } + { }10.0{\text{*P}}\left( {{\text{TM}}10.0} \right) + \cdots + 13.0{\text{*P}}\left( {{\text{TM}}13.0} \right)}}{{{\text{P}}\left( {{\text{TM}}9.5} \right){ } + {\text{ P}}\left( {{\text{TM}}10.0} \right) + \cdots + {\text{P}}\left( {{\text{TM}}13.0} \right)}},$$

in which, P(TM9.5) and so on represent the percentage of labeled IO neurons with TM9.5 averaged from all TM9.5 cases and so on.

## Supplementary Information

Supplementary Information.

## Data Availability

All data supporting the findings of this study are provided in the main text or supplementary information. This study did not generate a novel program code. This study did not generate new unique reagents. Requests for resources, datasets, protocols, and any other additional information should be directed to and will be fulfilled by the corresponding author, Izumi Sugihara (isugihara.phy1@tmd.ac.jp).

## Abbreviations

1+, 1−, 2+, 2−, 3+, 3−, 4+, 4−, 5+, 5−, 6+, 6−, 7+: Aldolase C (zebrin) stripes 1+, 1−, 2+, 2−, 3+, 3−, 4+, 4−, 5+, 5−, 6+, 6−, 7+
2b+, 4b+, 5a+: Aldolase C (zebrin) stripes 2b+, 4b+, 5a+
I–X: Lobules I–X
A, A2, AX, C1, C2, C3, CX, D0, D1, D2, X, X-CX: Modules A, A2, AX, C2, CX, D0, D1, D2, X, X-CX
a–c: Sublobules a–c (as in IXa–b)
arc: Arcuate nucleus
b+, c+, d+: Aldolase C (zebrin) stripes b+, c+, d+
beta: Subnucleus beta of the inferior olive
C: Caudal
cMAO: Caudal part of the medial accessory olive
cMAO-a: Subnucleus a of the cMAO
cMAO-b: Subnucleus b of the cMAO
cMAO-c: Subnucleus c of the cMAO
Cop: Copula pyramidis
Cr I: Crus I
Cr II: Crus II
D: Dorsal
DAO: Dorsal accessory olive
DC: Dorsal cap subnucleus (of the inferior olive)
DC/VLO: Dorsal cap and ventrolateral outgrowth subnuclei (of the inferior olive)
dDAO: Dorsal fold of the dorsal accessory olive
dPO: Dorsal lamella of the principal olive
DM: Dorsomedial subnucleus (of the inferior olive)
DMCC: Dorsomedial cell column subnucleus (of the inferior olive)
E: Embryonic day
e1+, e1−, e2−, f−: Aldolase C (zebrin) stripes e1+, e1−, e2−, f−
Fl: Flocculus
IO: Inferior olive
L: Lateral
Lt: Left
M: Medial
MAO: Medial accessory olive
N: Nodulus
P: Postnatal day
Par: Paramedian lobule
PC: Purkinje cell
PFl: Paraflocculus
PO: Principal olive
R: Rostral
rMAO: Rostral part of the medial accessory olive
Rt: Right
Sim: Simple lobule
TM: Tamoxifen injection at embryonic day, as in “TM11.5”
V: Ventral
vDAO: Ventral fold of the dorsal accessory olive
VLO: Ventrolateral outgrowth subnucleus (of the inferior olive)
vPO: Ventral lamella of the principal olive
