# Supplementary material for: Neurogenic timing of the inferior olive subdivisions is related to the olivocerebellar projection topography
Source: Sci Rep. 2023 May 2;13:7114. doi: 10.1038/s41598-023-33497-1 (PMC10154309; doi:10.1038/s41598-023-33497-1)
Supplement: Supplementary file 1 — Supplementary Information. [file 41598_2023_33497_MOESM1_ESM.docx]

**Supplementary information**

**Supplementary Table 1**. Specifications of used antibodies.

|  | Antigen | Immunogen | Manufacturer, species, mono- or polyclonal, catalog or lot No., RRID | Dilution used |
| --- | --- | --- | --- | --- |
| Primary antibodies | FoxP2 | Synthetic peptide derived from C-terminal amino acids of isoforms I, II, IV, and V of human FOXP2: REIEEEPLSEDLE | Everest Biotech, goat polyclonal, Cat.# EB05226, Lot # 160409, RRID: AB_2107112 | 1:5000 |
|  | FoxP2 | Full length protein corresponding to human FOXP2 | Abcam, mouse monoclonal, ab172320 | 1:1000 |
|  | Pcdh10 | The cytoplasmic domain of the OL-protocadherin isoform | Millipore, rat monoclonal, clone 5G10, Cat. # MABT20, Lot # NRG1759424, RRID:AB_10807416 | 1:1600 |
| Secondary antibodies | Anti-goat IgG, Alexa Fluor 488-conjugated | | Jackson ImmunoResearch, donkey, Cat. # 705-545-147 | 1:333 |
|  | Anti-mouse IgG, Alexa Fluor 647-conjugated | | Jackson ImmunoResearch, donkey, Cat. # 715-605-150 | 1:333 |
|  | Anti-rat IgG, Alexa Fluor 647-conjugated | | Jackson ImmunoResearch, donkey, Cat. # 712-605-150 | 1:333 |

**Supplementary Table 2.** The number of labeled neurons in the IO subdivisions in G2A::Ai9 mice with tamoxifen injection at different embryonic dates.

| Tamoxifen  timing | Mouse | Subdivisions | cMAO-a | cMAO-b | cMAO-c | Beta | DC/VLO | dDAO, medial | dDAO, lateral | vDAO, medial | vDAO, interm. | vDAO, lateral | dPO, medial | dPO, lateral | vPO, medial | vPO, lateral | DM | DMCC | rMAO | Total |
| --- | --- | --- | --- | --- | --- | --- | --- | --- | --- | --- | --- | --- | --- | --- | --- | --- | --- | --- | --- | --- |
| E9.5 | HG11b | Left | 0 | 0 | 0 | 0 | 0 | 0 | 0 | 0 | 0 | 0 | 0 | 0 | 0 | 0 | 0 | 0 | 0 | 0 |
|  |  | Right | 0 | 0 | 0 | 0 | 0 | 0 | 0 | 0 | 0 | 0 | 0 | 0 | 0 | 0 | 0 | 0 | 0 | 0 |
| E10.0 | HG07a | Left | 2 | 8 | 7 | 18 | 14 | 22 | 18 | 16 | 31 | 39 | 12 | 15 | 9 | 4 | 5 | 0 | 1 | 221 |
|  |  | Right | 3 | 9 | 7 | 24 | 26 | 25 | 13 | 31 | 45 | 57 | 20 | 27 | 4 | 9 | 8 | 1 | 3 | 324 |
|  | HG07b | Left | 1 | 3 | 8 | 15 | 15 | 24 | 12 | 35 | 19 | 27 | 15 | 10 | 10 | 4 | 3 | 1 | 2 | 204 |
|  |  | Right | 2 | 4 | 4 | 9 | 18 | 21 | 12 | 20 | 11 | 33 | 15 | 12 | 3 | 3 | 3 | 1 | 3 | 180 |
|  | HG36b | Left | 4 | 12 | 11 | 22 | 5 | 16 | 29 | 31 | 35 | 47 | 13 | 17 | 11 | 8 | 14 | 0 | 5 | 280 |
|  |  | Right | 0 | 17 | 22 | 25 | 12 | 12 | 21 | 42 | 36 | 62 | 21 | 21 | 15 | 3 | 14 | 2 | 6 | 331 |
| E10.5 | HG02a | Left | 10 | 47 | 52 | 97 | 53 | 40 | 53 | 93 | 134 | 134 | 80 | 95 | 22 | 28 | 68 | 7 | 24 | 1037 |
|  |  | Right | 12 | 41 | 71 | 92 | 50 | 62 | 59 | 127 | 107 | 124 | 61 | 105 | 29 | 33 | 53 | 11 | 31 | 1068 |
|  | HG02b | Left | 8 | 61 | 61 | 86 | 63 | 72 | 66 | 64 | 159 | 129 | 47 | 97 | 32 | 47 | 51 | 9 | 24 | 1076 |
|  |  | Right | 11 | 50 | 73 | 81 | 58 | 74 | 63 | 83 | 131 | 109 | 50 | 113 | 36 | 42 | 66 | 4 | 17 | 1061 |
|  | HG18a | Left | 17 | 45 | 48 | 82 | 40 | 52 | 65 | 87 | 78 | 104 | 51 | 79 | 33 | 37 | 53 | 6 | 40 | 917 |
|  |  | Right | 14 | 52 | 55 | 80 | 57 | 40 | 39 | 94 | 103 | 108 | 64 | 83 | 47 | 26 | 87 | 17 | 46 | 1012 |
| E11.0 | HG22a | Left | 40 | 134 | 121 | 107 | 65 | 44 | 49 | 151 | 141 | 101 | 100 | 167 | 69 | 66 | 89 | 24 | 92 | 1560 |
|  |  | Right | 39 | 128 | 117 | 110 | 64 | 48 | 47 | 141 | 111 | 109 | 83 | 149 | 61 | 78 | 106 | 15 | 54 | 1460 |
|  | HG04c | Left | 8 | 49 | 38 | 42 | 23 | 32 | 10 | 41 | 55 | 39 | 42 | 42 | 18 | 23 | 24 | 3 | 36 | 525 |
|  |  | Right | 6 | 30 | 39 | 36 | 34 | 31 | 20 | 45 | 60 | 42 | 29 | 34 | 22 | 8 | 40 | 4 | 21 | 501 |
|  | HG04d | Left | 40 | 121 | 117 | 95 | 65 | 19 | 39 | 115 | 90 | 82 | 103 | 162 | 51 | 57 | 131 | 23 | 87 | 1397 |
|  |  | Right | 56 | 115 | 83 | 84 | 54 | 22 | 19 | 76 | 68 | 88 | 72 | 110 | 41 | 43 | 124 | 15 | 107 | 1177 |
| E11.5 | HG03b | Left | 42 | 176 | 100 | 52 | 21 | 8 | 1 | 7 | 4 | 3 | 20 | 70 | 76 | 50 | 101 | 24 | 142 | 897 |
|  |  | Right | 58 | 226 | 165 | 53 | 16 | 0 | 0 | 17 | 8 | 4 | 36 | 77 | 92 | 56 | 131 | 26 | 156 | 1121 |
|  | HG03c | Left | 27 | 87 | 39 | 23 | 4 | 0 | 0 | 6 | 8 | 3 | 9 | 36 | 30 | 44 | 70 | 7 | 95 | 488 |
|  |  | Right | 27 | 91 | 39 | 23 | 4 | 0 | 0 | 6 | 8 | 3 | 9 | 36 | 30 | 44 | 70 | 7 | 95 | 492 |
|  | HG15d | Left | 61 | 123 | 90 | 59 | 14 | 1 | 0 | 18 | 10 | 10 | 31 | 59 | 35 | 46 | 95 | 13 | 107 | 772 |
|  |  | Right | 56 | 112 | 121 | 57 | 19 | 1 | 2 | 15 | 10 | 4 | 48 | 95 | 64 | 55 | 88 | 7 | 107 | 861 |
|  | HG09a | Left | 28 | 135 | 114 | 83 | 42 | 10 | 8 | 83 | 52 | 33 | 66 | 128 | 50 | 46 | 115 | 18 | 79 | 1090 |
|  |  | Right | 23 | 115 | 117 | 86 | 37 | 13 | 6 | 99 | 52 | 31 | 74 | 119 | 46 | 46 | 123 | 23 | 71 | 1081 |
| E12.0 | HG05a | Left | 5 | 2 | 0 | 0 | 0 | 0 | 0 | 0 | 0 | 0 | 0 | 0 | 0 | 1 | 2 | 0 | 15 | 25 |
|  |  | Right | 5 | 6 | 1 | 0 | 0 | 0 | 0 | 0 | 0 | 0 | 0 | 1 | 1 | 0 | 2 | 2 | 18 | 36 |
| E12.0 | HG05b | Left | 8 | 5 | 0 | 0 | 0 | 0 | 1 | 0 | 0 | 0 | 0 | 0 | 0 | 2 | 1 | 0 | 34 | 51 |
|  |  | Right | 13 | 9 | 2 | 0 | 0 | 2 | 0 | 0 | 0 | 0 | 0 | 0 | 0 | 1 | 1 | 0 | 13 | 41 |
|  | HG37a | Left | 0 | 1 | 2 | 2 | 0 | 0 | 0 | 1 | 0 | 0 | 1 | 0 | 0 | 0 | 1 | 0 | 1 | 9 |
|  |  | Right | 0 | 2 | 3 | 0 | 0 | 0 | 2 | 1 | 0 | 2 | 1 | 0 | 0 | 0 | 0 | 0 | 0 | 11 |
| E12.5 | HG01a | Left | 0 | 0 | 0 | 0 | 0 | 0 | 0 | 0 | 0 | 0 | 0 | 0 | 0 | 0 | 0 | 0 | 0 | 0 |
|  |  | Right | 0 | 0 | 0 | 0 | 2 | 0 | 0 | 0 | 0 | 0 | 0 | 0 | 0 | 0 | 0 | 0 | 0 | 2 |
|  | HG40a | Left | 0 | 0 | 0 | 0 | 0 | 0 | 0 | 0 | 0 | 0 | 0 | 0 | 0 | 0 | 0 | 0 | 0 | 0 |
|  |  | Right | 0 | 0 | 1 | 0 | 0 | 0 | 0 | 0 | 0 | 0 | 0 | 0 | 0 | 0 | 0 | 0 | 0 | 1 |
| E13.0 | HG08c | Left | 0 | 0 | 0 | 0 | 1 | 0 | 0 | 1 | 0 | 1 | 0 | 0 | 0 | 1 | 0 | 0 | 0 | 4 |
|  |  | Right | 0 | 1 | 0 | 0 | 0 | 0 | 0 | 0 | 0 | 0 | 0 | 0 | 0 | 0 | 0 | 0 | 0 | 1 |
| All IO Neurons | HG03b | Left | 582 | 1852 | 1003 | 701 | 472 | 400 | 245 | 974 | 837 | 714 | 585 | 848 | 649 | 405 | 1011 | 115 | 1328 | 12721 |
|  |  | Right | 749 | 1876 | 1381 | 694 | 383 | 427 | 177 | 826 | 999 | 620 | 607 | 576 | 685 | 343 | 990 | 133 | 1283 | 12749 |

Counted manually in serial sections immunostained for FoxP2. Tamoxifen injection at E13.5 did not produce any labeling in IO neurons.

**Supplementary Figure 1**.

**
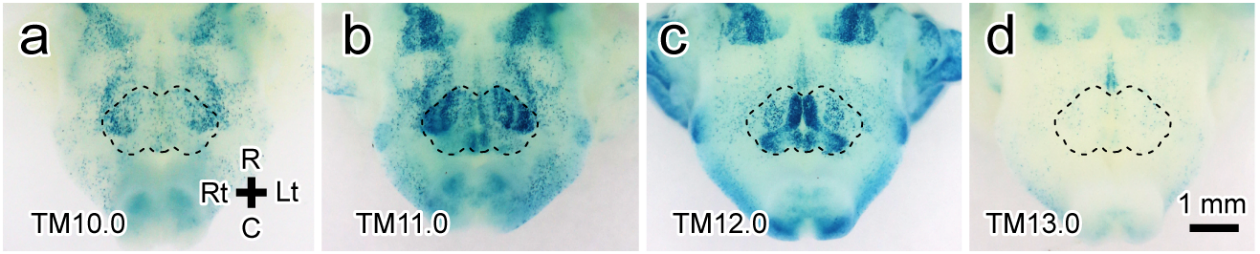
**

**Supplementary Figure 1**. Labeling of the inferior olive (IO) in G2A mice. Ventral view of the medulla of the whole mount preparation of the G2A::Tau^mGFP-nLacZ^ mouse at postnatal day (P) 7. Tamoxifen was given at E9.5 (a), E10.5 (b), E11.5 (c) and E12.5 (d). Dashed lines indicate the contour of the IO. Edited from Fig.2 of Hirata et al.[1]. See the separate list for abbreviations in the main manuscript. Created with Adobe Illustrator-10.3 and Adobe Photoshop-7.0.

**
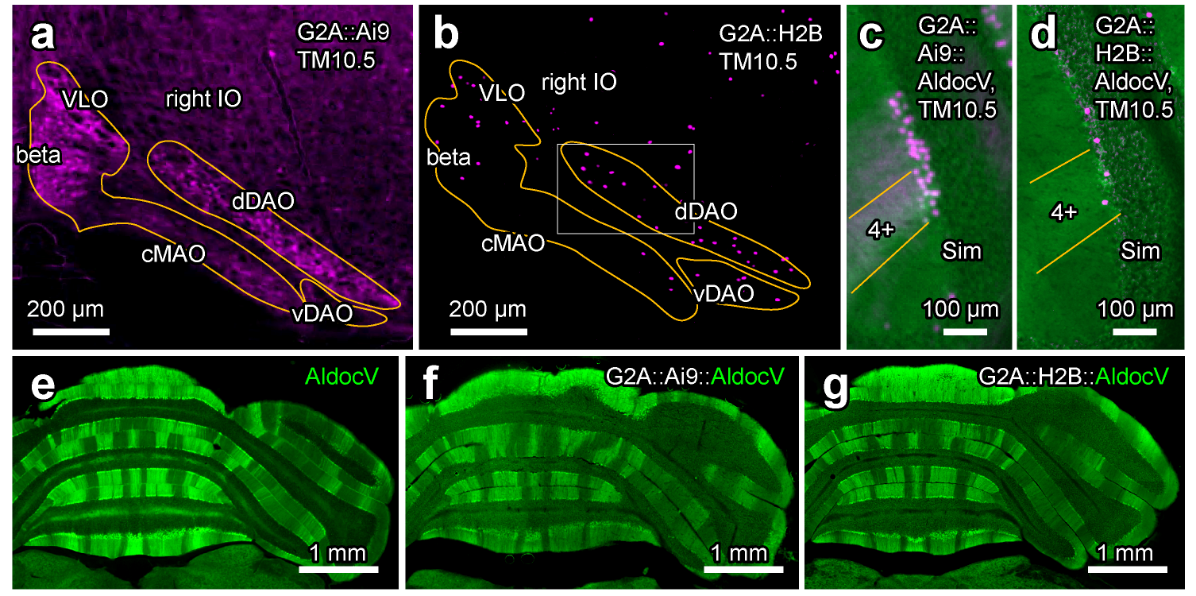
Supplementary Figure 2**.

**Supplementary Figure 2**. Sensitivity and specificity of R26R-H2B-mCherry (“H2B”) reporter mice. (a, b) Neuronal labeling in the right IO at the level of 40% from the caudal edge in coronal sections of G2A::Ai9::AldocV (a) and G2A::H2B (b) mice in which tamoxifen was given at E10.5. Yellow lines indicate the contour of the IO. White square indicates the area shown in Figure 1s. The tdTomato expression in Ai9 strain and the mCherry expression in H2B strain are shown in magenta pseudo color. (c, d) Purkinje cell labeling in the right simple lobule of the cerebellum in coronal sections of G2A::Ai9::AldocV (c) and G2A::H2B::AldocV (d) mice in which tamoxifen was given at E10.5. Yellow lines indicate boundaries of zebrin stripe 4+. The Venus signal is shown in green pseudo-color, whereas the tdTomato Ai9 reporter signal in the G2A::Ai9::AldocV mouse and the mCherry H2B reporter signal in the G2A::H2B::AldocV mouse are shown in magenta pseudo-color. (e-g) Zebrin stripes visualized with Venus expression in coronal sections at the caudal level of the cerebellum in the adult AldocV mouse (e), G2A::Ai9::AldocV mouse (f), and G2A::H2B::AldocV mouse (g). The pattern is nearly the same among these strains, suggesting the preservation of the normal cerebellar anatomy in these strains. See the separate list for abbreviations in the main manuscript. Created with Adobe Illustrator-10.3, Adobe Photoshop-7.0, and Zen 2.6.

**Supplementary Figure 3.**

**
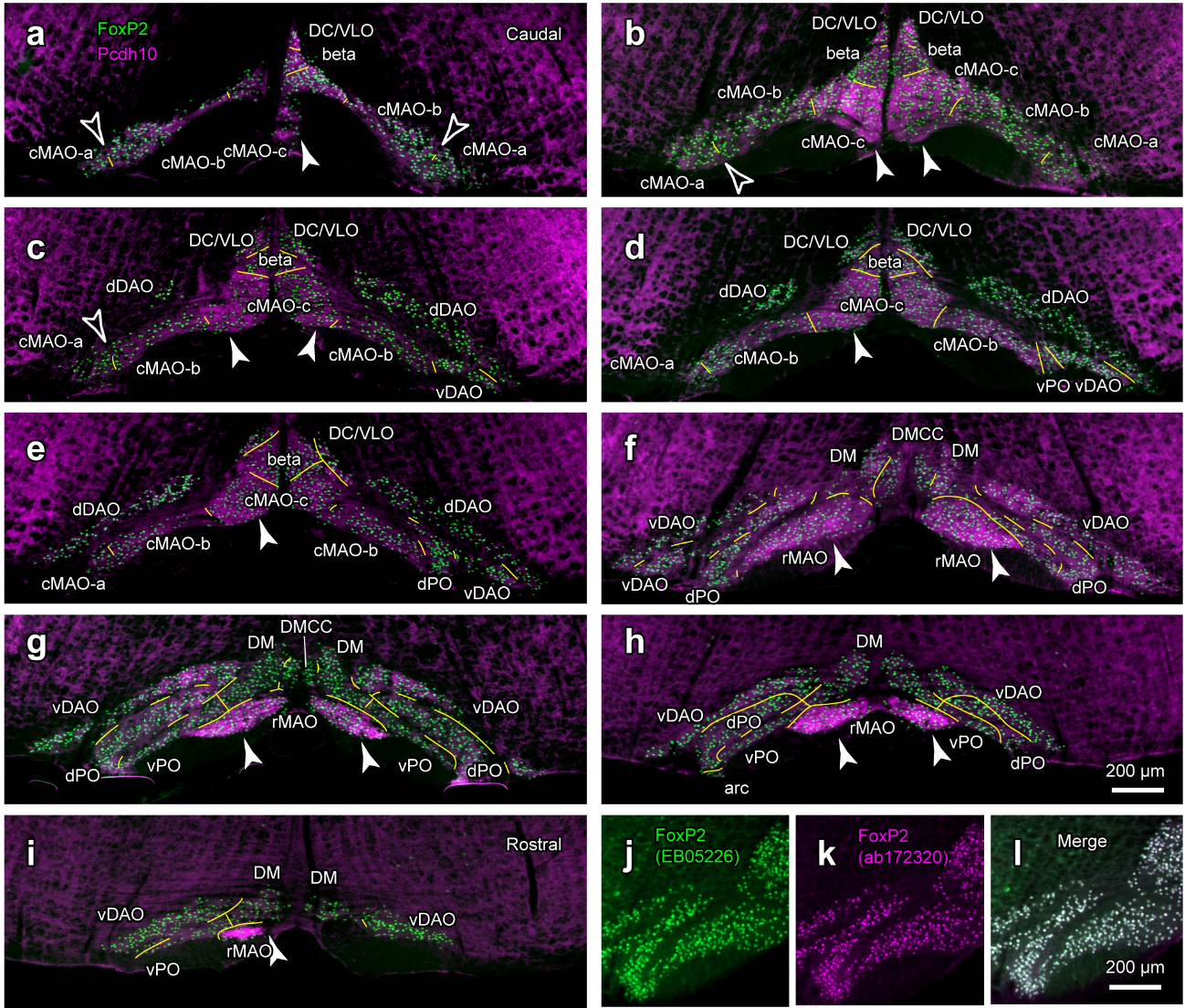
**

**Supplementary Figure 3.** Marker molecule expression patterns and subdivisions of the IO. (a–i) Coronal sections of the IO immunostained for FoxP2 (green) and Pcdh10 (magenta). Sections are from different caudorostral levels of the IO with an approximately 10% interval in an H2B::Ai9::AldocV mouse at P42. Filled arrowheads show areas with relatively higher Pcdh10 immunoreactivity. Open arrowheads show a gap of neuronal distribution at the putative boundary between cMAO-a and cMAO-b. (j–l) FoxP2 immunostaining with different antibodies (Everest goat antibody in green, Abcam mouse monoclonal antibody in magenta) producing virtually the same pattern. See the separate list for abbreviations in the main manuscript. Created with Adobe Illustrator-10.3, Adobe Photoshop-7.0, and Zen 2.6.

**Supplementary Figure 4**.


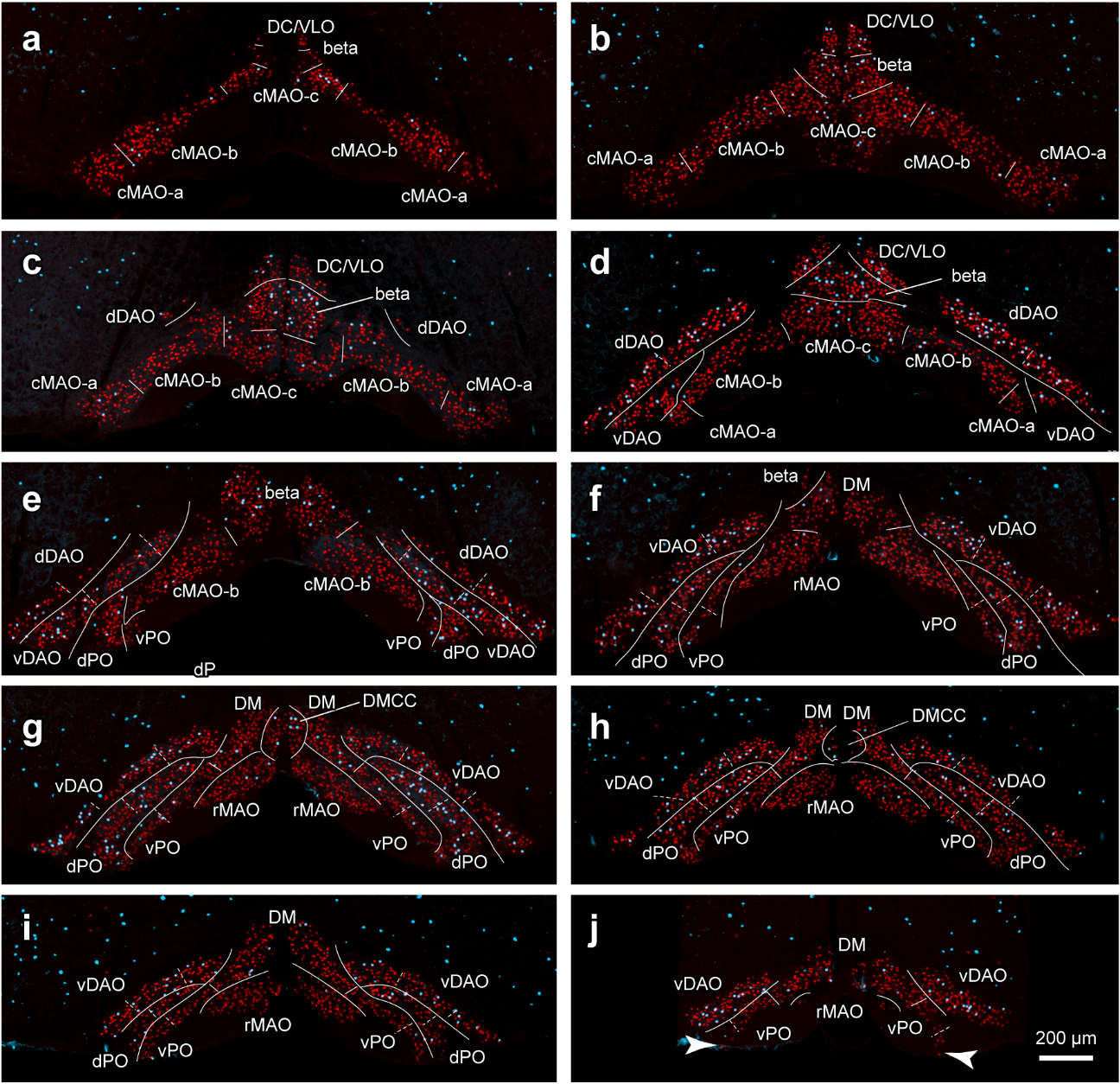


**Supplementary Figure 4**. Definition of IO subdivisions in the present study. (a–j) Coronal sections showing 10 different levels with an approximately 9% interval in a G2A::H2B mouse at P41. Signals indicate immunostaining for FoxP2 (red) and H2B reporter labeling (cyan, TM10.5). White lines indicate boundaries between IO subdivisions. See the separate list for abbreviations in the main manuscript. Created with Adobe Illustrator-10.3, Adobe Photoshop-7.0, and Zen 2.6.

References

1. Hirata, T. et al. NeuroGT: A brain atlas of neurogenic tagging CreER drivers for birthdate-based classification and manipulation of mouse neurons. *Cell. Rep. Methods.* **1(3)**:100012 (2021). doi: 10.1016/j.crmeth.2021.100012.
